# Integrated smart farm system for *Centella asiatica* L. cultivation: development and assessment of multi-tier cultivation effects on bioactive compound production

**DOI:** 10.3389/fpls.2025.1646310

**Published:** 2025-09-08

**Authors:** Hyeonbyeong Lee, Jeonghyun Baek, Donghyuk Im, Jeongwook Heo, Taehyun Kim

**Affiliations:** Smart Farm Development Division, Department of Agricultural Engineering, National Institute of Agricultural Sciences, Rural Development Administration, Jeollabuk-do, Republic of Korea

**Keywords:** *Centella asiatica*, smart farm, hydroponics, controlled environment agriculture, bioactive compounds, DFT system, medicinal plants, year-round production

## Abstract

**Introduction:**

Traditional soil-based cultivation of Centella asiatica L. faces seasonal limitations and quality inconsistency.

**Methods:**

This study developed and assessed a smart farm system incorporating Deep Flow Technique (DFT) hydroponics for stable, year-round production with consistent bioactive compound profiles. System development was conducted at the National Institute of Agricultural Sciences, followed by a six-month field assessment at Chungju Agricultural Technology Center, South Korea. The cultivation system comprised a 99 m² greenhouse with a 19.2 m² active hydroponic area, maintaining key environmental parameters.

**Results:**

We observed differential bioactive compound accumulation between cultivation tiers. Madecassic acid content was 2.3-fold higher in the LED-equipped tier (1.25 ± 0.04 mg/g) versus the fluorescent-equipped tier (0.54 ± 0.03 mg/g, p<0.001).

**Discussion:**

The system demonstrated operational reliability, enabling year-round production and establishing a technological foundation for controlled environment medicinal plant cultivation with standardized compound profiles.

## 1 Introduction

*Centella asiatica* L., a perennial herb in the Apiaceae family, has a long history of traditional use as both a medicinal and edible crop throughout Asia. Its major bioactive components—triterpenes including asiaticoside, madecassoside, asiatic acid, and madecassic acid—are extensively utilized in pharmaceuticals and cosmetics due to their anti-inflammatory, wound-healing, and collagen-synthesis-promoting properties (Chandrika and Prasad Kumara, 2015). The pharmacological significance of *C. asiatica* continues to be underscored by recent research, with studies reporting its extract’s acetylcholinesterase inhibitory activity, antioxidant effects (Hafiz and Bhat, 2020), and cognitive function enhancement (Sbrini et al., 2020). A systematic review further corroborated the wound healing efficacy of *C. asiatica* extracts (Arribas-López et al., 2022), highlighting its versatile application as a raw material in diverse medicinal and cosmetic products (Wang et al., 2024; Witkowska et al., 2024; Kwon et al., 2012; Diniz et al., 2023; Gray et al., 2018).

Predominantly thriving in tropical and subtropical climates, *C. asiatica* is widely distributed in regions like India and Indonesia, while in South Korea, its natural habitat is confined to southern islands such as Jeju. This abundance of functional compounds positions *C. asiatica* as a crucial raw material within the pharmaceutical and cosmetic sectors (Alqahtani et al., 2015; Vélez-Terreros et al., 2024); however, traditional cultivation methods struggle to consistently meet the quality and quantity demands of these industries. Despite increasing demand in Korea, *C. asiatica* production relies on conventional soil cultivation, which presents several constraints. Soil cultivation restricts the growing season to June–October, making a stable year-round supply challenging. Furthermore, the heterogeneity of soil environments leads to significant variations in functional compound content, complicating standardization. These practical constraints create a clear need for alternative cultivation systems that can provide consistent, year-round production with standardized quality profiles.

Hydroponics offers a technological solution to address these limitations. Specifically, the Deep Flow Technique (DFT) hydroponic method is well-established for improving crop productivity (Hu et al., 2008; Soussi et al., 2024; Liu et al., 2020). Recent advances in Information and Communication Technology (ICT) and the adoption of smart farm systems facilitate precise monitoring and control over crop cultivation environments. Research on *C. asiatica* hydroponics has investigated the impact of environmental variables on functional compound levels, such as using fungal elicitors (Prasad et al., 2013) or altering nutrient solution electrical conductivity (EC) (Shawon et al., 2023). While these studies demonstrate feasibility, they often lack comprehensive system integration and extended field validation.

Current smart farm research emphasizes the integration and deployment of advanced technologies for practical agricultural applications (Said Mohamed et al., 2021; Navarro et al., 2020). The application of AI and big data is receiving increased attention (Idoje et al., 2021; Akkem et al., 2023; Wolfert et al., 2017), and smart farming is seen as a key element for sustainable agricultural development through digitalization and automation (Walter et al., 2017; Javaid et al., 2022). However, practical challenges remain in system integration and enhancing farmer capabilities (Pivoto et al., 2017; de Souza et al., 2022). A significant gap exists between individual technology components and complete, field-validated production systems (Schukat and Heise, 2021; Haque et al., 2021; Amiri-Zarandi et al., 2022b, a).

The contribution of this study lies in the systematic integration and comprehensive field validation of hydroponic and smart farm technologies specifically tailored for medicinal plant production, with *Centella asiatica* L. serving as a model crop. Our work advances the field by demonstrating how integrated precision agriculture systems can be optimized for bioactive compound production. Building upon established technologies, we developed and validated a fully integrated DFT hydroponic system with tailored environmental controls through comprehensive pilot-scale trials. The primary objectives were: (1) To develop and assess an integrated smart farm system for stable, year-round production of *C. asiatica*; (2) To assess bioactive compound accumulation patterns under different cultivation tier conditions, while acknowledging the inherent confounding factors; (3) To demonstrate the system’s operational feasibility through extended field trials; and (4) To provide performance data and protocols to establish a technical foundation for controlled environment medicinal plant cultivation.

## 2 Materials and methods

### 2.1 Experimental materials

*Centella asiatica* L. plants (‘Good Byungpul’ and ‘Giant’ cultivars), initially propagated at a commercial farm in Chungju, Chungcheongbuk-do, Korea, served as the experimental material. Plants exhibiting uniform growth characteristics under preliminary soil cultivation were selected for the hydroponic experiments. The hydroponic facility was established within a 13×8 m (approximately 99 m^2^) singlespan plastic greenhouse at the National Institute of Agricultural Sciences, Korea. The facility included a urethane-coated floor, shade screens, ventilation fans, humidifiers, and an appropriately sized electric air conditioning unit for year-round temperature regulation.

### 2.2 Experimental system construction

#### 2.2.1 Hydroponic system

**Figure 1:**
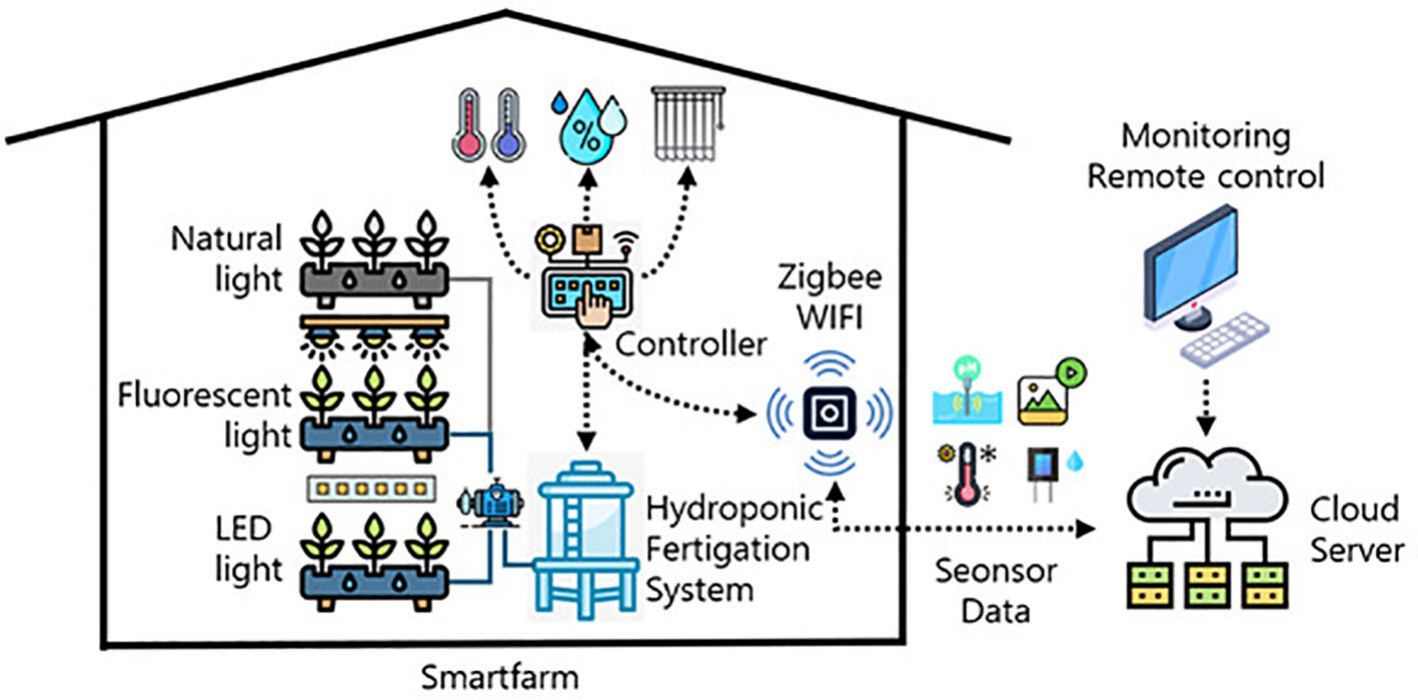
Overall structural diagram of the Deep Flow Technique (DFT) hydroponic smart farm system showing the multi-tier cultivation setup with nutrient circulation and environmental control components.

The core of the system was a multi-tier Deep Flow Technique (DFT) setup (see **Figure 1**). The system was designed with a flexible multi-tier configuration. For the present study, experiments were conducted using two tiers (19.2 m^2^ active cultivation area) to ensure optimal environmental control and uniform experimental conditions across all cultivation units. Each tier comprised a 1.2 m × 8 m cultivation bed (9.6 m^2^ per tier). Beds were fabricated from stainless steel square tubing with uniformly NC-machined perforations. Nutrient solution circulation was facilitated by waterproof liners forming channels within each bed. Expanded polystyrene (EPS) boards (1200×1000×150 mm) fitted with planting panels held the plants.

**Figure 2:**
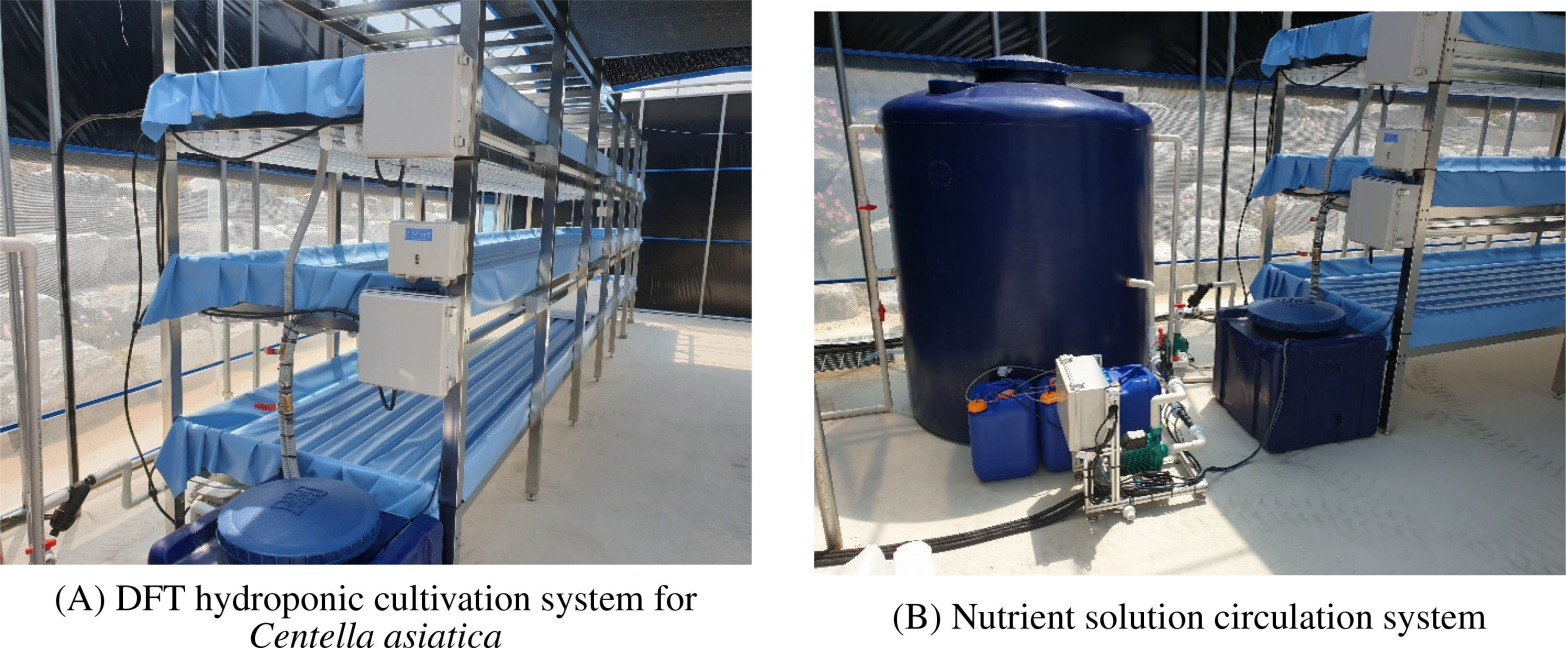
Implementation of the *Centella asiatica* smart farm prototype showing **(A)** the DFT hydroponic cultivation system and **(B)** the nutrient circulation infrastructure.

**Figure 2** provides an overview of the constructed smart farm system layout, showing the DFT hydroponic cultivation system for *Centella asiatica* (**Figure 2A**) and the nutrient solution circulation system (**Figure 2B**).

The DFT approach maintained a constant nutrient solution level within the beds. Adjustable drainage outlets controlled the maximum solution depth, while pumps automatically replenished the solution as levels decreased. A compressor delivered air through six one-way valves per bed to ensure adequate dissolved oxygen levels. Operation of the compressor and artificial lights was managed by timer controllers. The nutrient delivery subsystem included three stock solution tanks (A, B, C), a raw water tank, two return tanks, and a nutrient solution chiller. Standard leafy vegetable nutrient formulations (e.g., Mulpurie) were used for solutions A and B, with diluted nitric acid in tank C for pH adjustment. Solution delivery was automated based on target EC and pH setpoints. An external chiller regulated nutrient solution temperature, particularly during warmer months. Complete nutrient solution replacement occurred monthly. Initial root growth was encouraged by providing only pH-adjusted water for approximately two weeks post-transplanting before introducing the full nutrient solution. EC and pH sensors were calibrated annually.

#### 2.2.2 Environmental control system

Environmental monitoring relied on various sensors. An EC sensor (0–20 mA output corresponding to 0.0–20.0 dS/m) and a pH sensor (0–20 mA output for 0.0–14.0 pH) monitored the nutrient solution. Substrate water content sensors (0–100% VWC range) and air temperature/humidity sensors (-40°C to +60°C range) monitored the root zone and aerial environments, respectively. A central controller featuring a 16-bit MCU managed data acquisition and system actuation. It featured 8 general-purpose input/output (GPIO) ports and communicated via RS-232 and RS-485 protocols.

**Figure 3:**
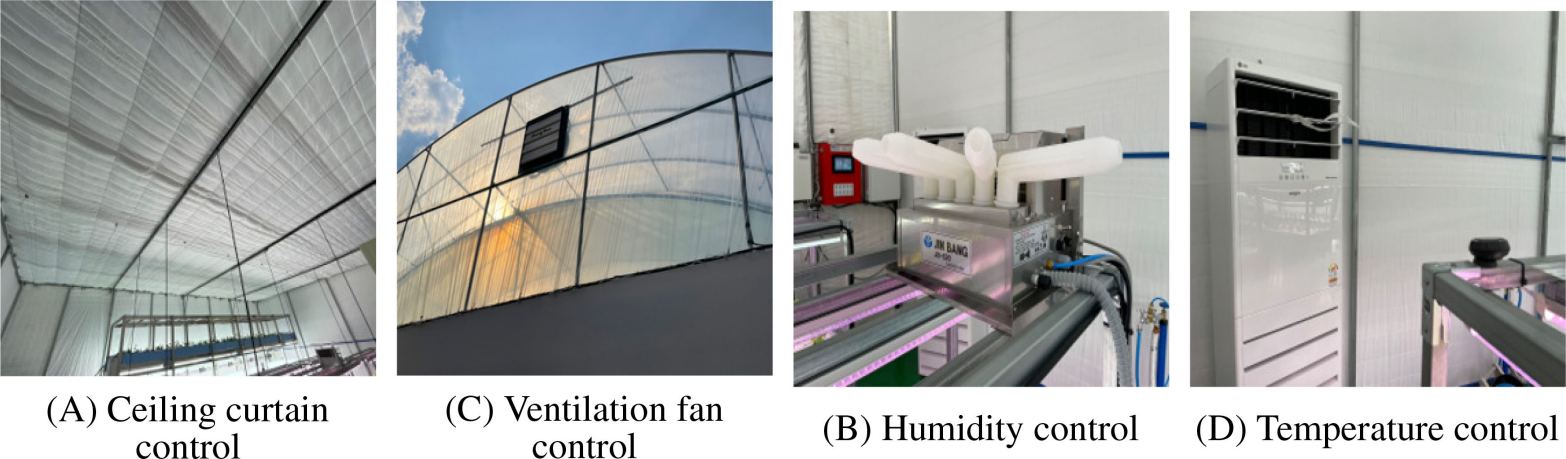
Environmental control systems of the smart farm greenhouse showing various automated control mechanisms: **(A)** Ceiling curtain control for light management, **(B)** Humidity control device, **(C)** Ventilation fan for air circulation, and **(D)** Temperature control unit.

The environmental control system, as shown in **Figure 3**, included automated management of ceiling curtains for light regulation, humidity control through humidification systems, ventilation fan control for air circulation, and temperature control via heating and cooling systems. These integrated control mechanisms ensured optimal environmental conditions for *Centella asiatica* cultivation throughout the growing period.

#### 2.2.3 Lighting system

To assess potential effects of different lighting systems in the multi-tier configuration, the first (lower) tier was equipped with 22W bar-type LEDs, while the second (upper) tier was equipped with 32W tri-phosphor fluorescent lamps. It is crucial to acknowledge that this experimental configuration places different lighting treatments on different tiers, which creates an inherent confounding between lighting type and tier position. This design characteristic represents a fundamental limitation that prevents the definitive isolation of lighting effects from other tier-related micro-environmental factors (e.g., temperature, air circulation gradients). Therefore, observed differences between tiers must be interpreted as the combined influence of lighting characteristics and tier-specific conditions, not as the effect of lighting alone. Future studies would benefit from randomized tier assignments or controlled positioning strategies to de-confound these variables. Lights were arranged in 6 rows, with a total of 12 lamp units per tier (i.e., 2 lamps per row), to promote uniform irradiance. Light intensity measurements confirmed spatial uniformity, with deviations within ±4.5% of the mean value across measurement points. Detailed specifications are provided in **Table 1**.

**Table 1:** Specifications of the monitoring system and cultivation beds.

Category	Component	Specification
Sensor	EC	0–20 mA
	pH	0–14 pH
Controller	CPU	16-bit MCU
	Output port	GP 8-port
	Input port	GP 8-port
	Communication port	RS-232, RS-485
Monitoring Panel	OS	Windows or Linux
	Size	8 inch
	Communication port	RS-232, RS-485 or Ethernet
Cropgrowing facility	Facility Structure	Stainless steel angled tube
	LED	Bar type 22W
	Fluorescent light	32W
	EPS	1200×1000×150 mm
	Pot & Plate	2000×30,000 mm

#### 2.2.4 Light spectral characteristics

The LED lighting system employed broad-spectrum white LEDs (22W Bar-type, commercial LED manufacturer) with the following approximate spectral distribution: blue (400–500 nm): 25%, green (500600 nm): 30%, red (600–700 nm): 35%, far-red (700–800 nm): 10%. Light intensity was maintained at 90–95 *µ*mol·m^−2^·s^−1^ Photosynthetic Photon Flux Density (PPFD) with a 16:8 hour photoperiod. Standard tri-phosphor fluorescent lamps (32W) provided broader wavelength distribution across the visible range (400–700 nm) with relatively uniform spectral output. The LED system exhibited approximately 30% higher photosynthetic photon efficacy (PPE) compared to the fluorescent lighting, based on power consumption and PPFD measurements.

### 2.3 Cultivation conditions and experimental design

#### 2.3.1 Nutrient solution management

A standard hydroponic nutrient formulation was employed based on consultation with local agricultural technology centers. Target parameters were: solution temperature 20 ± 1°C (maintained as a critical requirement), EC 0.9 ± 0.1 dS/m, and pH 5.6 ± 0.2. Dissolved oxygen was maintained above 20 ppm via continuous aeration using bubble generators. Pathogen control was assisted by a solution sterilizer. Daily monitoring and adjustments ensured stable solution quality.

#### 2.3.2 Environmental control and data acquisition

**Figure 4:**
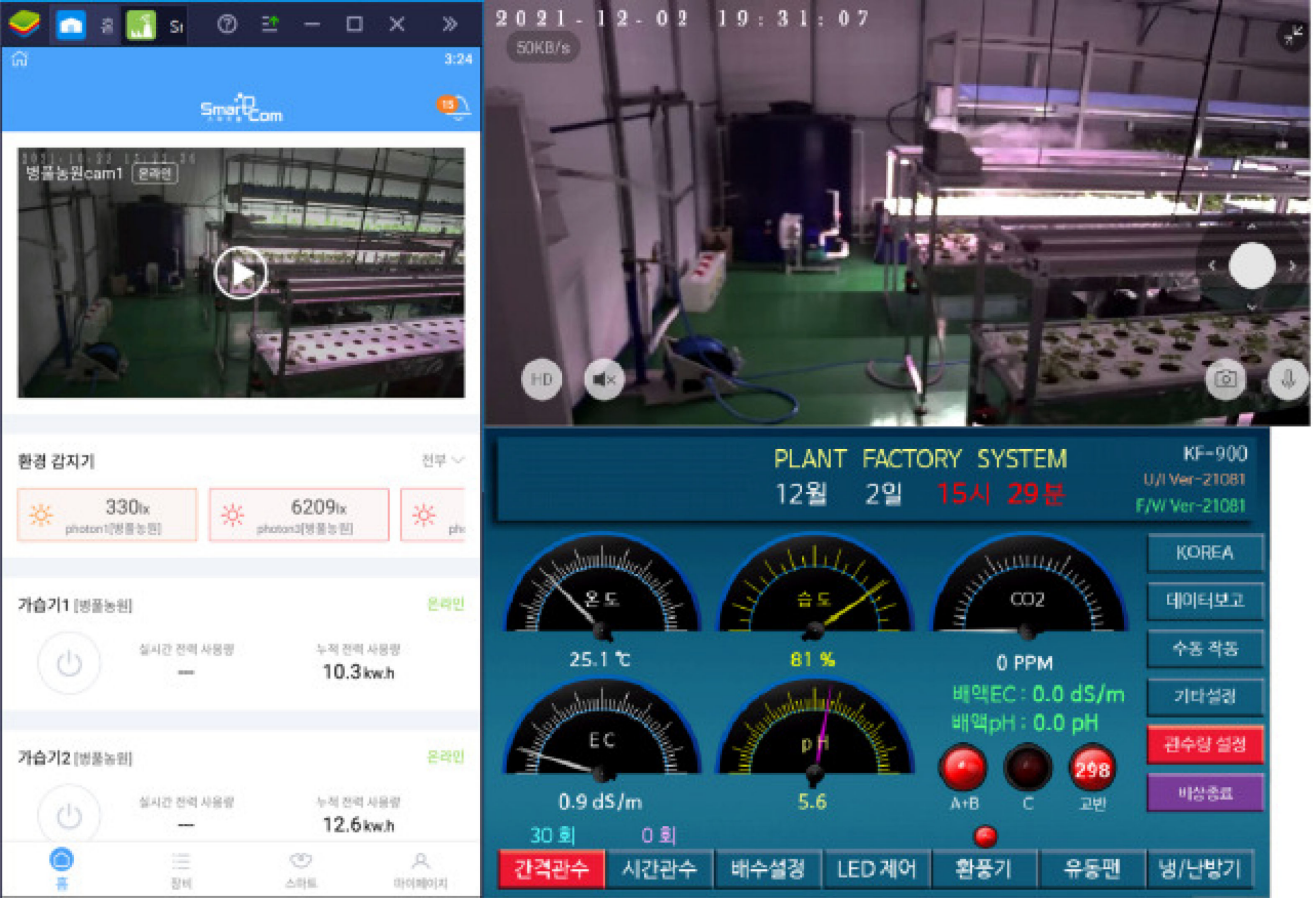
User interface of the remote monitoring and control system showing real-time environmental parameters, sensor data visualization, and automated control status for the integrated smart farm system.

The aerial environment was precisely controlled with target settings of: daytime temperature 25 ± 2°C, nighttime temperature 15 ± 2°C, relative humidity 60 ± 5%, light intensity 6,700–7,000 Lux (equivalent to 90–95 *µ*mol·m^−2^·s^−1^ Photosynthetic Photon Flux Density, PPFD) and CO_2_ concentration 800 ± 100 ppm. Real-time monitoring and automated adjustments were performed by the control system via Zigbee and WiFi communication. A relational database management system (RDBMS) was implemented for comprehensive data logging and analysis. Sensor data (temperature, humidity, light intensity, CO_2_) and visual data from PTZ and fixed cameras were collected. Actuator status data (humidifier, air conditioner, vents, fans, air curtain) were also logged. MariaDB and MySQL were utilized for data storage and management. A REST API facilitated real-time data exchange between the server and monitoring/control hardware, enabling efficient operation (see **Figure 4**). Cloud integration enhanced system stability and data accessibility.

#### 2.3.3 Soil cultivation for comparison

For comparison, soil-grown *C. asiatica* plants were cultivated concurrently. These plants were grown in 15-cm diameter pots filled with a commercial nursery potting mix (Baroker, Seoul Bio, Eumseong, Korea). The pots were placed within the same greenhouse facility to expose them to similar ambient temperature and humidity conditions, but they received only natural sunlight filtered through the greenhouse plastic. Plants were watered daily to maintain adequate soil moisture.

#### 2.3.4 Bioactive compound analysis

At 15 weeks post-transplanting, when the plant canopy had fully developed, the aerial parts of the plants were harvested for bioactive compound analysis. Functional compounds (madecassoside, asiaticoside, madecassic acid, asiatic acid) were quantified using High-Performance Liquid Chromatography (HPLC; Agilent 1260 Infinity II, Agilent Technologies, CA, USA) equipped with a ZORBAX Eclipse Plus C18 column (4.6 × 100 mm, 3.5 m). Samples were prepared by drying harvested leaves at 60°C for 72 hours, grinding them into a fine powder, and extracting 0.1 g of powder with 10 mL of methanol via sonication for 60 minutes. The extract was filtered through a 0.45 m syringe filter prior to analysis. The mobile phase consisted of 0.1% phosphoric acid in water (A) and acetonitrile (B). The gradient elution was as follows: 0–10 min, 25-35% B; 10–20 min, 35-65% B; 20–22 min, 65-25% B; 22–25 min, 25% B. The flow rate was 1.0 mL/min, the injection volume was 10 L, the column temperature was maintained at 30°C, and detection was performed at 210 nm. Compounds were identified by comparing retention times with authentic standards (Sigma-Aldrich, St. Louis, MO, USA).

#### 2.3.5 Statistical analysis

The experiment was laid out using three replicate blocks to account for potential spatial variation within the greenhouse. Within each block, the cultivation system consisted of a multi-tier unit where lighting treatments (LED and fluorescent) were assigned to fixed tier positions. We acknowledge that this fixed assignment does not constitute a fully randomized design regarding the lighting treatment. Transplanting occurred on June 10, 2022. Cuttings were placed into individual cups. For root initiation, stem bases were submerged in pH-adjusted water for two weeks. Subsequently, the full nutrient solution was introduced. Growth assessments were conducted over six months. From each treatment, ten plants were randomly sampled periodically across the three blocks to measure leaf length, leaf width, leaf count, fresh weight, dry weight, and root growth parameters. Statistical analyses were performed using SPSS 26.0 (IBM Corp., Armonk, NY, USA). Normality of data distribution was assessed using the Shapiro-Wilk test. When normality assumptions were met, independent t-tests compared means between two groups. For multi-group comparisons (demonstration farm), one-way ANOVA followed by Duncan’s Multiple Range Test (DMRT) was used. Significance levels were set at p ¡ 0.05. Results are presented as mean ± standard error of the mean (SEM). Sample sizes were determined based on available greenhouse space and resource constraints, with n=10 plants per treatment group. We acknowledge that this sample size provides limited statistical power, and future studies would benefit from larger sample sizes to enhance the robustness of the conclusions.

## 3 Results

### 3.1 Environmental control performance

#### 3.1.1 Light environment

Throughout the cultivation period, light intensity measurements confirmed stable conditions within the target range of 6,700–7,000 Lux (90–95 *µ*mol·m^−2^·s^−1^) for both LED and fluorescent treatments. Spatial uniformity analysis across six points per tier indicated deviations within ±4.5% of the mean, ensuring a consistent light environment.

#### 3.1.2 Temperature and humidity

The smart farm control system successfully maintained air temperatures at 25 ± 2°C (day) and 15 ± 2°C (night), and relative humidity around 60 ± 5%. This stable control effectively mitigated the extreme temperature fluctuations often encountered in traditional soil cultivation during summer and winter.

#### 3.1.3 Nutrient solution environment

The DFT system provided stable control over key nutrient solution parameters. Temperature was maintained at 20 ± 1°C, EC at 0.9 ± 0.1 dS/m, and pH at 5.6 ± 0.2. Continuous aeration ensured dissolved oxygen levels remained above 20 ppm, fostering a favorable root-zone environment.

### 3.2 Plant growth progression

**Figure 5:**
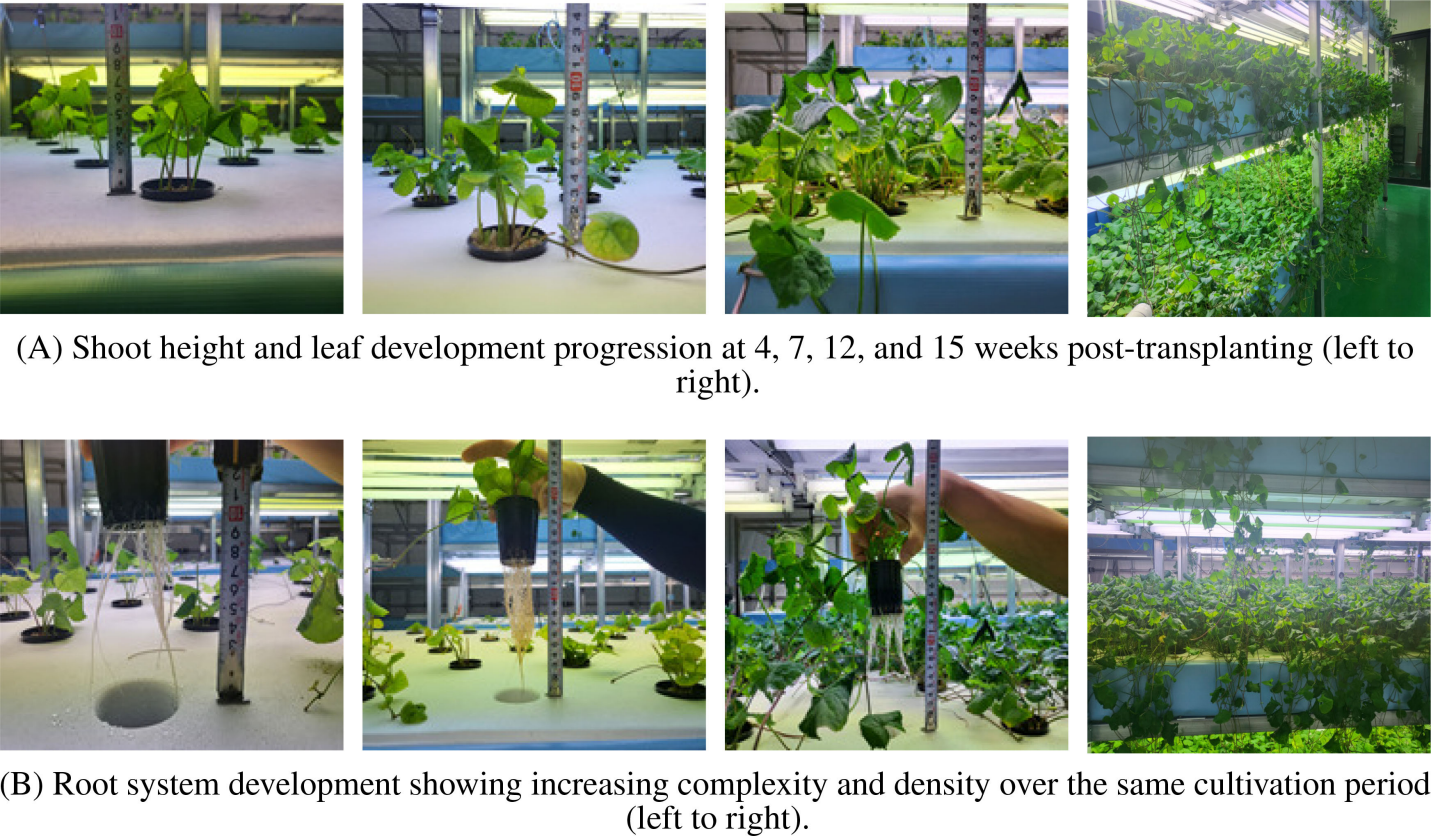
Growth progression of *C. asiatica* post-transplanting. **(A)** Shoot height and leaf development progression at 4, 7, 12, and 15 weeks (left to right). **(B)** Root system development over the same period (left to right).

The temporal growth pattern of *C. asiatica* post-transplanting is illustrated in **Figure 5**. Key observations include:

**Week 4:** Initial establishment phase. Roots reached 100 mm in length, appearing healthy white. Shoot height was 75 mm, leaves were dark green, and lateral stem elongation commenced.
**Week 7:** Accelerated vegetative growth. Root length increased to 130 mm, shoot height to 110 mm, accompanied by prolific new leaf development.
**Week 12:** Maturation phase. Root elongation slowed (stabilizing around 135 mm), but lateral root proliferation intensified. Shoot height reached 150 mm. Continuous lateral growth led to inter-plant contact.
**Week 15:** Dense canopy formation. Vigorous lateral expansion resulted in full coverage of the cultivation panel.

### 3.3 Bioactive compound content analysis

The bioactive compound contents of *C. asiatica* under various cultivation conditions are presented in **Table 2**.

**Table 2:** Comparison of bioactive compound content (mg/g dry weight) in *C. asiatica* under different cultivation conditions and tier positions.

Cultivation System	Light Source/Tier Position	Madecassoside	Asiaticoside	Madecassic acid	Asiatic acid
National Institute of Agricultural Sciences (NAS) Experiments					
DFT Hydroponics	LED-equipped tier	2.63 ± 1.18^ns^	52.08 ± 1.18^***^	1.25 ± 0.04^***^	0.81 ± 0.03^***^
DFT Hydroponics	Fluorescent-equipped tier	2.12 ± 0.98	40.90 ± 1.49	0.54 ± 0.03	0.51 ± 0.02
Demonstration Farm Experiments					
DFT Hydroponics	Fluorescent (1st floor, Bottom)	1.84 ± 0.48^a^	31.76 ± 0.32^b^	0.86 ± 0.04^c^	0.89 ± 0.08^b^
DFT Hydroponics	Fluorescent (2nd floor, Middle)	2.11 ± 0.72^a^	43.48 ± 1.12^a^	1.33 ± 0.04^b^	1.10 ± 0.03^a^
DFT Hydroponics	Fluorescent (3rd floor, Top)	2.10 ± 0.54^a^	41.79 ± 1.49^a^	1.51 ± 0.01^a^	1.19 ± 0.04^a^
Comparison with Soil Cultivation					
Soil Cultivation	Natural/Ambient Light	2.92 ± 1.08^ns^	67.64 ± 1.82^***^	0.46 ± 0.04^*^	0.60 ± 0.01^**^
DFT Hydroponics	Fluorescent-equipped tier	2.12 ± 0.98	40.90 ± 1.49	0.54 ± 0.03	0.51 ± 0.02

Values are mean ± SEM (n=10). For NAS experiments, asterisks denote significant differences between LED and fluorescent tiers based on independent t-tests (***p¡0.001; ns, not significant). For Demonstration Farm, means within a column followed by the same letter are not significantly different (DMRT, p¿0.05). For Soil vs. Hydroponics, asterisks denote significance based on independent t-tests (*p¡0.05, **p¡0.01, ***p¡0.001).

#### 3.3.1 Tier-based cultivation comparison (NAS experiments)

Comparing cultivation tiers, madecassoside content did not differ significantly between the LED-equipped and fluorescent-equipped tiers (2.63 ± 1.18 mg/g vs. 2.12 ± 0.98 mg/g; *t*(18) = 0.576*,p* = 0.572). However, asiaticoside content was significantly higher in the LED-equipped tier (52.08 ± 1.18 mg/g) compared to the fluorescent-equipped tier (40.90 ± 1.49 mg/g; *t*(18) = 10.196*,p <* 0.001). Similarly, madecassic acid content was 2.3-fold higher in the LED-equipped tier (1.25 ± 0.04 mg/g) than in the fluorescentequipped tier (0.54 ± 0.03 mg/g; *t*(18) = 24.857*,p <* 0.001). Asiatic acid content was also 1.6-fold higher in the LED-equipped tier (0.81 ± 0.03 mg/g) versus the fluorescent-equipped tier (0.51 ± 0.02 mg/g; *t*(18) = 12.786*,p <* 0.001). Again, we emphasize that these differences reflect the combined effects of tier-specific cultivation conditions rather than isolated lighting effects.

#### 3.3.2 Effect of tier position in multi-tier system (demonstration farm experiments)

At the demonstration farm, the position of cultivation tiers influenced bioactive compound levels. Madecassoside content did not vary significantly across the three tiers (1st floor: 1.84 ± 0.48 mg/g; 2nd floor: 2.11 ± 0.72 mg/g; 3rd floor: 2.10 ± 0.54 mg/g; DMRT, p¿0.05). However, for asiaticoside, the 2nd (43.48 ± 1.12 mg/g) and 3rd (41.79 ± 1.49 mg/g) tiers showed significantly higher content (DMRT, p¡0.05) than the 1st (bottom) tier (31.76 ± 0.32 mg/g). Madecassic acid content increased with tier height, being lowest on the 1st floor (0.86 ± 0.04 mg/g), intermediate on the 2nd (1.33 ± 0.04 mg/g), and highest on the 3rd (top) tier (1.51 ± 0.01 mg/g; DMRT, p¡0.05). A similar trend was observed for asiatic acid, with the 2nd (1.10 ± 0.03 mg/g) and 3rd (1.19 ± 0.04 mg/g) tiers showing higher content (DMRT, p¡0.05) than the 1st tier (0.89 ± 0.08 mg/g).

#### 3.3.3 Cultivation method comparison

When comparing compound profiles between cultivation methods, plants grown in the fluorescentequipped hydroponic tier showed significant differences compared to soil-grown plants. For example, asiaticoside content was significantly lower in fluorescent hydroponics (40.90 ± 1.49 mg/g) compared to soil cultivation (67.64 ± 1.82 mg/g; *t*(18) = −19.692*,p <* 0.001). Conversely, madecassic acid content was significantly higher in fluorescent hydroponics (0.54 ± 0.03 mg/g) than in soil cultivation (0.46 ± 0.04 mg/g; *t*(18) = 2.807*,p* = 0.012). Asiatic acid content was lower in fluorescent hydroponics (0.51 ± 0.02 mg/g) compared to soil cultivation (0.60 ± 0.01 mg/g; *t*(18) = −5.592*,p <* 0.001).

## 4 Discussion

### 4.1 System performance and integration

The developed smart farm-based DFT hydroponic system integrates established technologies for controlled environment medicinal plant production. The six-month field assessment provides robust evidence of the system’s technical feasibility. Its operational reliability was demonstrated by its ability to consistently maintain key environmental parameters within tight target ranges (e.g., solution temperature at 20 ± 1°C and EC at 0.9 ± 0.1 dS/m) despite external climate fluctuations.

The experimental configuration placed different lighting systems on different tiers, which inevitably introduced environmental variations beyond lighting conditions alone. This design characteristic represents a fundamental limitation that prevents the definitive isolation of lighting effects from other tier-related micro-environmental factors. Therefore, our findings should be interpreted as reflecting the performance of the entire tier setup (i.e., position plus lighting) rather than the effect of the light source alone. Future studies designed to isolate specific environmental effects would require careful control of individual variables through randomized positioning or controlled environmental chambers.

### 4.2 Interpretation of tier-based bioactive compound variations

The differential bioactive compound accumulation patterns observed between cultivation tiers provide insights into environmental influences on secondary metabolite production in controlled environments. The enhanced accumulation of triterpene acids (madecassic acid, asiatic acid) in upper tiers suggests a potential shift in metabolic pathways. This could be linked to subtle environmental stressors associated with the upper tier position, such as potentially higher temperatures or altered air circulation, which have been reported to influence secondary metabolite production in other plant species (e.g., Ramakrishna and Ravishankar, 2011).

The variation in compound profiles between different experimental locations (NAS vs. Demonstration Farm) reflects the influence of local environmental conditions, seasonal timing, and facility-specific factors on plant metabolism. These variations highlight both the challenges and opportunities in controlled environment agriculture, where environmental standardization becomes critical for consistent production outcomes.

From a practical perspective, the observed tier-based variations suggest that cultivation systems could potentially be optimized for specific compound profiles depending on intended end-use applications. However, such optimization would require systematic investigation of individual environmental factors and their interactions to establish evidence-based cultivation protocols.

### 4.3 Practical applications and system benefits

The integrated smart farm system addresses critical challenges in medicinal plant production that directly impact commercial viability. The demonstrated year-round production capability represents a significant advancement over traditional seasonal cultivation limitations. Key practical advantages include: (1) elimination of seasonal production constraints enabling continuous supply for pharmaceutical applications; (2) standardized environmental control reducing batch-to-batch variation in compound profiles; (3) reduced post-processing requirements through elimination of soil contamination; and (4) modular design facilitating scalable implementation across different facility configurations.

The successful collaboration with Chungju Agricultural Technology Center demonstrates the system’s practical applicability and acceptance by agricultural extension services, indicating readiness for broader implementation. The system’s validated performance also opens opportunities for collaborative research with pharmaceutical or cosmetic industry partners to tailor compound profiles for specific product pipelines.

### 4.4 Study limitations and future research directions

This study has several limitations that provide direction for future investigations. The tier-based experimental design, while providing valuable operational data, prevents definitive attribution of compound differences to specific environmental factors. Future research priorities should therefore focus on: (1) controlled comparative studies using a randomized block design to isolate individual environmental effects, such as light spectrum and intensity; (2) mechanistic investigations into the environmental regulation of triterpene biosynthesis pathways, for example, through transcriptomic (RT-qPCR) analysis of key genes in response to specific environmental cues; (3) optimization studies for targeted compound production based on these mechanistic insights; and (4) comprehensive economic feasibility assessments for commercial-scale implementation.

Additionally, expanding the system validation to other high-value medicinal plants would enhance the broader applicability of the developed technologies. Generalizability across different climates and plant species will require further validation.

### 4.5 Conclusions

This research establishes a technological foundation for precision medicinal plant cultivation, addressing critical gaps between laboratory-scale studies and commercial-scale production. The six-month field assessment demonstrates the system’s operational feasibility while providing important baseline data for future optimization. This study addresses a real-world challenge by offering a pathway to achieving a reliable supply of high-quality raw materials for the pharmaceutical and cosmetic industries.

Key contributions include: (1) demonstration of stable environmental control in multi-tier hydroponic systems; (2) documentation of tier-based bioactive compound variation; (3) systematic integration of precision agriculture technologies for medicinal plants; and (4) establishment of performance metrics for scaled development.

The study provides a technical framework for addressing industry requirements for a consistent, yearround raw material supply. While acknowledging the methodological limitations in isolating specific environmental effects, the demonstrated system performance establishes feasibility for commercial-scale development and identifies concrete research priorities for continued optimization, such as isolating lighting effects and conducting economic modeling. The integration of established technologies into a comprehensive, field-assessed system is a necessary step in translating research into practical agricultural applications, contributing to sustainable medicinal plant production.

## Data Availability

The raw data supporting the conclusions of this article will be made available by the authors, without undue reservation.
